# Supplementary material for: Efficacy and safety of oral Chinese medicine on cancer-related fatigue for lung cancer patients after chemotherapy: Protocol for systematic review and meta-analysis
Source: PLoS One. 2022 Jun 30;17(6):e0270203. doi: 10.1371/journal.pone.0270203 (PMC9246193; doi:10.1371/journal.pone.0270203)
Supplement: S2 Table — (PDF) [file pone.0270203.s002.pdf]

Measures of CRF

Note : Scales should be required to quantify the presence and severity of CRF.

Brief Fatigue Inventory (BFI)

Cancer-Related Fatigue Distress Scale (CRFDS)

Cancer Fatigue Scale (CFS)

Chalder Fatigue Scale (CFQ)

Daily Fatigue Cancer Scale

European Organization for Research and Treatment of Cancer-Fatigue (EORTC-Fatigue)

Edmonton Symptom Assessment Scale (ESAS)

Fatigue Symptom Inventory (FSI)

Fatigue Assessment Questionnaire (FAQ)

Functional Assessment of Cancer Therapy-Fatigue (FACT-F)

Functional Assessment of Chronic Illness Therapy-Fatigue(FACIT-F)

MD Anderson Symptom Inventory-Fatigue item (MDASI-F)

Multidimensional Fatigue Symptom Inventory (MFSI)

Multidimensional Fatigue Inventory (MFI)

Profile of Mood States-Fatigue Scale (POMS-F)

Piper Fatigue Scale (PFS)

Tang Fatigue Rating Scale (TFRS)

Visual Analog Fatigue Scale (VAS-F)

Schwartz Cancer Fatigue Scale
