# Supplementary material for: Efficacy and safety of oral Chinese medicine on cancer-related fatigue for lung cancer patients after chemotherapy: Protocol for systematic review and meta-analysis
Source: PLoS One. 2022 Jun 30;17(6):e0270203. doi: 10.1371/journal.pone.0270203 (PMC9246193; doi:10.1371/journal.pone.0270203)
Supplement: S1 File — (PDF) [file pone.0270203.s003.pdf]

Search strategies for databases

Embase

#1 'chemotherapy-related fatigue':ti,ab,kw OR 'cancer related fatigue':ti,ab,kw OR fatigue:ti,ab,kw OR 'chemotherapy-induced fatigue':ti,ab,kw OR 'CRF':ti,ab,kw

#2 'cancer related fatigue'/exp OR 'fatigue'/exp

#3 #1~#2/OR

#4 'lung neoplasms':ti,ab,kw OR 'lung neoplasm':ti,ab,kw OR 'neoplasm, lung':ti,ab,kw OR 'pulmonary neoplasm':ti,ab,kw OR 'neoplasms, pulmonary':ti,ab,kw OR 'neoplasm, pulmonary':ti,ab,kw OR 'lung cancer':ti,ab,kw OR 'lung cancers':ti,ab,kw OR 'cancer, lung':ti,ab,kw OR 'cancers, lung':ti,ab,kw OR 'cancer of lung':ti,ab,kw OR 'cancer of the lung':ti,ab,kw OR 'pulmonary cancer':ti,ab,kw OR 'pulmonary cancers':ti,ab,kw OR 'cancer, pulmonary':ti,ab,kw OR 'cancers, pulmonary':ti,ab,kw

#5 'lung cancer'/exp OR 'lung adenoma'/exp

#6 #4~#5/OR

#7 'medicine, chinese traditional':ti,ab,kw OR 'traditional chinese medicine':ti,ab,kw OR 'traditional chinese medicines':ti,ab,kw OR 'chinese medicines':ti,ab,kw OR 'traditional medicine, chinese':ti,ab,kw OR 'chinese traditional medicine':ti,ab,kw OR 'chinese medicine, traditional':ti,ab,kw OR 'drugs, chinese herbal':ti,ab,kw OR 'chinese herbal drugs':ti,ab,kw OR 'herbal drugs, chinese':ti,ab,kw OR 'herbal medicine':ti,ab,kw OR 'herbal medicines':ti,ab,kw OR 'medicine, herbal':ti,ab,kw OR herb:ti,ab,kw OR herbs:ti,ab,kw

#8 'Chinese drug'/exp OR 'Chinese medicine'/exp OR 'herbal medicine'/exp

#9 #7~#8/OR

#10 'crossover procedure':de OR 'double-blind procedure':de OR 'randomized controlled trial':de OR 'single-blind procedure':de OR (random* OR factorial* OR crossover* OR cross NEXT/1 over* OR placebo* OR doubl* NEAR/1 blind* OR singl* NEAR/1 blind* OR assign* OR allocat* OR volunteer*):de,ab,ti

#11 #3 AND #6 AND #9 AND #10

Ovid

#1 (chemotherapy-related fatigue or cancer related fatigue or fatigue or chemotherapy-induced fatigue or CRF).tw.

#2 (lung neoplasms or lung neoplasm or neoplasm, lung or pulmonary neoplasm or neoplasms, pulmonary or neoplasm, pulmonary or lung cancer or lung cancers or cancer, lung or cancers, lung or cancer of lung or cancer of the lung or pulmonary cancer or pulmonary cancers or cancer, pulmonary or cancers, pulmonary).tw.

#3 (medicine, chinese traditional or traditional chinese medicine or traditional chinese medicines or chinese medicines or traditional medicine, chinese or chinese traditional medicine or chinese medicine, traditional or drugs, chinese herbal or chinese herbal drugs or herbal drugs, chinese or herbal medicine or herbal medicines or medicine, herbal or herb or herbs).tw.

#4 (randomized controlled trial or controlled clinical trial).pt,mt. or randomized.ab. or placebo.ab. or drug therapy.fs. or randomly.ab. or trial.ab. or groups.ab.

#5 exp animals/ not humans.sh.

#6 #4 NOT #5

#5 #1 AND #2 AND #3 AND #6

Web of Science search strategy:

Search in: All Databases(Web of Science Core Collection, Korean Journal Database, MEDLINE, SciELO Citation Index)

#1 ((((TS=(cancer related fatigue)) OR TS=(fatigue)) OR TS=(CRF)) OR TS=(chemotherapy-induced fatigue)) OR TS=(chemotherapy-related fatigue)

#2 ((((((((((((((((TS=(Lung Neoplasms)) OR TS=(Lung Neoplasm)) OR TS=(Neoplasm, Lung)) OR TS=(Neoplasm, Lung)) OR TS=(Pulmonary Neoplasm)) OR TS=(Neoplasms, Pulmonary)) OR TS=(Neoplasm, Pulmonary)) OR TS=(Lung Cancer)) OR TS=(Lung Cancers)) OR TS=(Cancer, Lung)) OR TS=(Cancers, Lung)) OR TS=(Cancer of Lung)) OR TS=(Cancer of the Lung)) OR TS=(Pulmonary Cancer)) OR TS=(Pulmonary Cancers)) OR TS=(Cancer, Pulmonary)) OR TS=(Cancers, Pulmonary)

#3 ((((((((((((((TS=(Medicine, Chinese traditional)) OR TS=(Traditional Chinese Medicine)) OR TS=(Traditional Chinese Medicines)) OR TS=(Traditional Chinese Medicines)) OR TS=(Chinese medicines)) OR TS=(Traditional Medicine, Chinese)) OR TS=(Chinese Traditional Medicine)) OR TS=(Chinese Medicine, Traditional)) OR TS=(Drugs, Chinese Herbal)) OR TS=(Chinese Herbal Drugs)) OR TS=(Herbal Drugs, Chinese)) OR TS=(Herbal Medicine)) OR TS=(Herbal Medicines)) OR TS=(Medicine, Herbal)) OR TS=(Herb)) OR TS=(Herbs)

#4 ((((((TS=(Randomized Controlled Trial)) OR TS=(randomized controlled trial)) OR TS=(randomised controlled trial)) OR TS=(randomized controlled trials)) OR TS=(randomised controlled trials)) OR TS=(Clinical trials, Randomized)) OR TS=(clinical trial)

#5 #1 AND #2 AND #3 AND #4

CENTRAL search strategy:

#1 (chemotherapy-related fatigue):ti,ab,kw OR (cancer related fatigue):ti,ab,kw OR (fatigue):ti,ab,kw OR (chemotherapy-induced fatigue):ti,ab,kw OR (CRF):ti,ab,kw

#2 MeSH descriptor: [Fatigue] this term only

#3 MeSH descriptor: [Fatigue Syndrome, Chronic] this term only

#4 #1~#3/OR

#5 (Lung Neoplasms):ti,ab,kw OR (Lung Neoplasm):ti,ab,kw OR (Neoplasm, Lung):ti,ab,kw OR (Neoplasm, Lung):ti,ab,kw OR (Neoplasms, Pulmonary):ti,ab,kw

#6 (Pulmonary Neoplasm):ti,ab,kw OR (Neoplasm, Pulmonary):ti,ab,kw OR (Lung Cancer):ti,ab,kw OR (Lung Cancers):ti,ab,kw OR (Cancer, Lung):ti,ab,kw

#7 (Cancers, Lung):ti,ab,kw OR (Cancer of Lung):ti,ab,kw OR (Cancer of the Lung):ti,ab,kw OR (Pulmonary Cancer):ti,ab,kw OR (Pulmonary Cancers):ti,ab,kw

#8 (Cancer, Pulmonary):ti,ab,kw OR (Cancers, Pulmonary):ti,ab,kw

#9 MeSH descriptor: [Lung Neoplasms] this term only

#10 MeSH descriptor: [Carcinoma, Non-Small-Cell Lung] this term only

#11 MeSH descriptor: [Small Cell Lung Carcinoma] this term only

#12 #5~#11/OR

#13 (Medicine, Chinese traditional):ti,ab,kw OR (Traditional Chinese Medicine):ti,ab,kw OR (Traditional Chinese Medicines):ti,ab,kw OR (Traditional Chinese Medicines):ti,ab,kw OR (Traditional Medicine, Chinese):ti,ab,kw

#14 (Chinese medicines):ti,ab,kw OR (Chinese Traditional Medicine):ti,ab,kw OR (Chinese Medicine, Traditional):ti,ab,kw OR (Drugs, Chinese Herbal):ti,ab,kw OR (Chinese Herbal Drugs):ti,ab,kw

#15 (Herbal Drugs, Chinese):ti,ab,kw OR (Herbal Medicines):ti,ab,kw OR (Medicine, Herbal):ti,ab,kw OR (Herb):ti,ab,kw OR (Herbs):ti,ab,kw

#16 MeSH descriptor: [Medicine, Chinese traditional] this term only

#17 MeSH descriptor: [Herbal Medicine] this term only

#18 #13~#17/OR

#19 #4 AND #12 AND #18

CNKI search strategy:

(SU=疲乏 OR SU=疲劳 OR SU=癌因性疲乏 OR SU=癌性疲乏 OR SU=癌症相关性疲乏 OR SU=化疗疲乏) AND (SU=肺癌 OR SU=肺恶性肿瘤 OR SU=肺肿瘤 OR SU=小细胞肺癌 OR SU=非小细胞肺癌) AND (SU=中药 OR SU=中草药 OR SU=草药 OR SU=中医) AND (SU=随机 OR FT=随机)

Wan Fang search strategy:

主题:(疲乏+疲劳+癌因性疲乏+癌性疲乏+癌症相关性疲乏+化疗疲乏)*主题:(肺癌+肺恶性肿瘤+肺肿瘤+小细胞肺癌+非小细胞肺癌)*主题:(中药+中草药+草药+中医)*随机

SinoMed search strategy:

#1 疲乏 OR 癌因性疲乏 OR 癌性疲乏 OR 癌症相关性疲乏 OR 化疗疲乏

#2 主题词=疲劳/全部副主题词

#3 主题词=精神疲劳/全部副主题词

#4 主题词=疲劳综合征，慢性/全部副主题词

#5 #1~#4/OR

#6 肺癌 OR 肺恶性肿瘤 OR 肺肿瘤 OR 小细胞肺癌 OR 非小细胞肺癌

#7 主题词=肺肿瘤/全部副主题词

#8 主题词=小细胞肺癌/全部副主题词

#9 主题词=癌，非小细胞肺/全部副主题词

#10 #6~#9/OR

#11 中药 OR 中草药 OR 草药 OR 中医

#12 主题词=中药疗法/全部副主题词

#13 主题词=中医疗法/全部副主题词

#14 主题词=中草药/全部副主题词

#15 主题词=草药医学/全部副主题词

#16 #11~#15/OR

#17 随机 OR 盲法 OR 安慰剂

#18 主题词=随机对照试验[文献类型]

#19 主题词=随机分配

#20 主题词=随机对照试验/全部副主题词

#21 #17~#20/OR

#22 #5 AND #10 AND #16 AND #21

VIP search strategy:

M=(疲乏+疲劳+癌因性疲乏+癌性疲乏+癌症相关性疲乏+化疗疲乏)*M=(肺癌+肺恶性肿瘤+肺肿瘤+小细胞肺癌+非小细胞肺癌)*M=(中药+中草药+草药+中医)*U=随机

ClinicalTrials.gov

#1 cancer related fatigue OR chemotherapy-related fatigue OR fatigue OR chemotherapy-induced fatigue (Condition or disease)

#2 traditional chinese medicine OR traditional chinese medicines OR chinese traditional medicine OR chinese herbal drugs OR herbal medicine OR herbal medicines OR herb OR herbs (Intervention/treatment)

#3 #1 AND #2

International Clinical Trials Registry Platform of WHO(ICTRP)

#1 cancer related fatigue OR chemotherapy-related fatigue OR fatigue OR chemotherapy-induced fatigue [Title]

#2 lung neoplasms OR lung neoplasm OR lung cancer OR lung cancers OR pulmonary cancer OR pulmonary cancers [Condition]

#3 traditional chinese medicine OR traditional chinese medicines OR chinese traditional medicine OR chinese herbal drugs OR herbal medicine OR herbal medicines OR herb OR herbs [Intervention]

#4 #1 AND #2 AND #3
